# Impact of Peer Review in the Radiation Treatment Planning Process: Experience of a Tertiary Care University Hospital in Pakistan

**DOI:** 10.1200/JGO.19.00039

**Published:** 2019-08-08

**Authors:** Bilal Mazhar Qureshi, Muhammad Atif Mansha, Muneeb Uddin Karim, Asim Hafiz, Nasir Ali, Benazir Mirkhan, Fatima Shaukat, Maria Tariq, Ahmed Nadeem Abbasi

**Affiliations:** ^1^ The Aga Khan University, Karachi, Pakistan

## Abstract

**PURPOSE:**

To evaluate and report the frequency of changes in radiation therapy treatment plans after peer review in a simulation review meeting once a week.

**MATERIALS AND METHODS:**

Between July 1 and August 31, 2016, the radiation plans of 116 patients were discussed in departmental simulation review meetings. All plans were finalized by the primary radiation oncologist before presenting them in the meeting. A team of radiation oncologists reviewed each plan, and their suggestions were documented as no change, major change, minor change, or missing contour. Changes were further classified as changes in clinical target volume, treatment field, or dose. All recommendations were stratified on the basis of treatment intent, site, and technique. Data were analyzed by Statistical Package for the Social Sciences and are presented descriptively.

**RESULTS:**

Out of 116 plans, 26 (22.4%) were recommended for changes. Minor changes were suggested in 15 treatment plans (12.9%) and a major change in 10 (8.6%), and only one plan was suggested for missing contour. The frequency of change recommendations was greater in radical radiation plans than in palliative plans (92.3% *v* 7.7%). The head and neck was the most common treatment site recommended for any changes (42.3%). Most of the changes were recommended in the technique planned with three-dimensional conformal radiation therapy (50%). Clinical target volume (73.1%) was identified as the most frequent parameter suggested for any change, followed by treatment field (19.2%) and dose (0.08%).

**CONCLUSION:**

Peer review is an important tool that can be used to overcome deficiencies in radiation treatment plans, with a goal of improved and individualized patient care. Our study reports changes in up to a quarter of radiotherapy plans.

## INTRODUCTION

Ensuring a high level of clinical quality in the practice of radiation oncology has become increasingly important. The advancing knowledge about diseases and the increasing complexity of radiation planning and delivery methods can make radiation therapy vulnerable to errors.^1^ A considerable variation in target volume delineation among radiation oncologists may occur, which can influence local tumor control and can result in undesirable complications in patients.^2^ These differences exist despite adherence to well-established organ dose criteria and uniform contouring guidelines.^3^ Therefore, a process is required to prevent inadequacies in contouring and radiation treatment planning.

Quality assurance (QA) is a systematic way of determining inadequacies in any process by meeting a required set of specifications or expectations. A QA program provides a unified set of quality indicators that focus on machines, personnel, and patients to ensure safe and effective use of radiation therapy.^4^ Peer review is an important measure for QA in radiation oncology.^5^ It has been defined as “the evaluation of creative work or performance by other people in the same field to enhance the quality of work or the performance of colleagues.”^6^ A meta-analysis has shown that an audit and feedback can be effective in improving professional practice.^7^ However, there is a dearth of quality data in this regard in the field of radiation oncology, and guidelines regarding peer review with specific recommendations are few.^8^

Recognizing the need for peer review in the radiation therapy treatment planning process, a simulation review meeting (SRM) was held once a week in the radiation oncology department starting from 2006 to December 2016. All patients for whom radiation therapy was being planned were discussed routinely in the SRM after undergoing the initial planning processes of contouring and field arrangements. The SRM included a review of the planned treatment by all the radiation oncologists, and changes were recommended and made as a result of peer review, with consensus. The objective of this study was to evaluate whether peer review in weekly SRMs influences the radiation therapy treatment planning process.

## MATERIALS AND METHODS

This study was conducted from July 1 to August 31, 2016, after approval by the institutional ethical review committee. Patients who were booked for external beam radiation therapy and underwent simulation were discussed routinely in the weekly SRM of the radiation oncology section of Aga Khan University (AKU) Hospital. All patients, regardless of intent of treatment or technique of delivering radiotherapy, were included in the study. Before discussion in the SRM, the contouring of target volume was completed and approved by the primary consultant radiation oncologist. Each plan was reviewed by at least two or three radiation oncologists. All modifications were categorized as no change, minor change, major change, or missing contour (Table 1). Each change was further classified as change in clinical target volume (CTV), treatment field, or dose. Modification frequency was analyzed chronologically and by treatment intent, tumor site, and treatment technique. Intent of treatment was recorded as either curative or palliative. Tumor sites included the brain, head and neck, thorax, abdomen, pelvis, extremity, and others. Treatment techniques were two dimensional, three-dimensional conformal radiation therapy (3D-CRT), and intensity modulated radiation therapy (IMRT). Data were analyzed by Statistical Package for the Social Sciences version 23 (SPSS, Chicago, IL) and are presented descriptively.

**TABLE 1:**
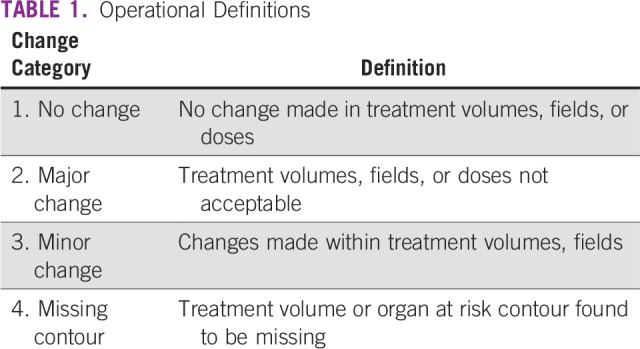
Operational Definitions

## RESULTS

The study was conducted for a period of 2 months, during which the radiation treatment plans of 116 patients with cancer were reviewed in the weekly SRM. A median of three consultant radiation oncologists attended the SRM, with a range of two to four, together with radiation oncology residents and medical physicists.

**FIG 1:**
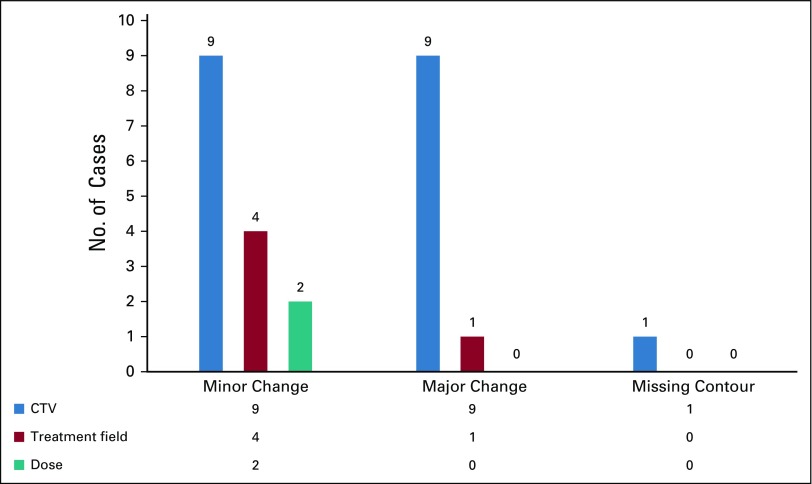
Classification of changes. CTV, clinical target volume.

Out of 116 patients, 96 (82.8%) were planned to be treated with curative intent, and 20 (17.2%) were planned to be treated with palliative intent. Head and neck (n = 47 [40.5%]) was the most common reviewed site, followed by the thorax (n = 30 [25.9%]) and pelvis (n = 17 [14.7%]). The most frequent technique used in treatment planning was 3D-CRT, which was used in 71 cases (61.2%), followed by IMRT (n = 30 [25.9%]). A total of 26 changes (22.6%) were recommended in radiation treatment plans. Minor changes were recommended in 15 reviewed cases (12.9%), whereas a major change was recommended in 10 cases (8.6%; Table 2). Most changes were suggested in CTV (n = 19 [73.1%]), with the least changes suggested in the prescribed dose (n = 2 [0.08%]). Additional classification of changes with respect to CTV, treatment field, and dose is presented in Fig 1.

**TABLE 2:**
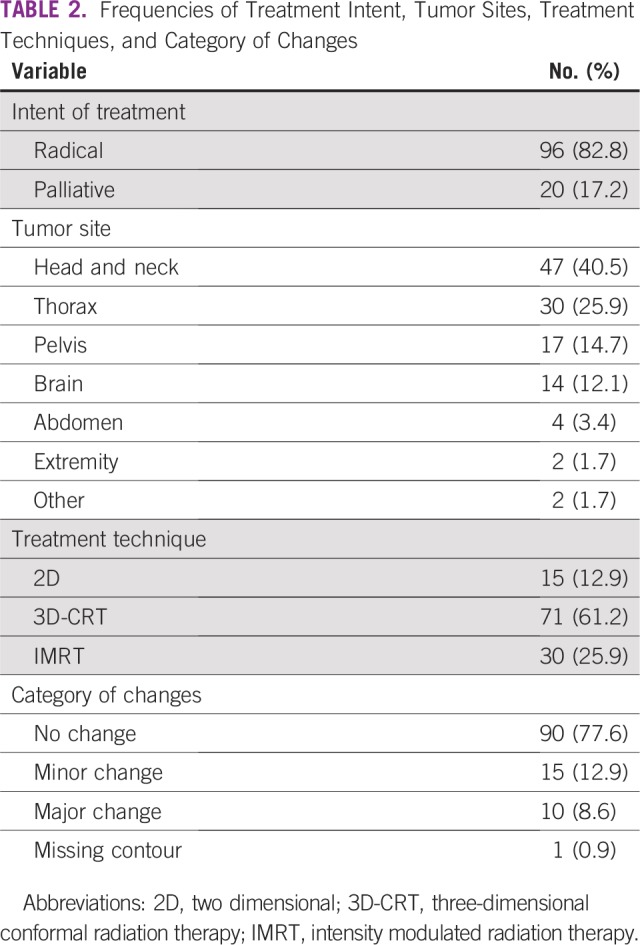
Frequencies of Treatment Intent, Tumor Sites, Treatment Techniques, and Category of Changes

**FIG 2:**
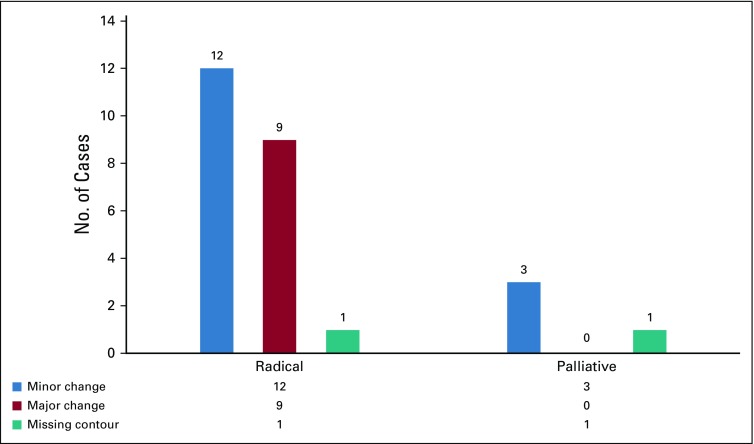
Categorization of changes with respect to treatment intent.

**FIG 3:**
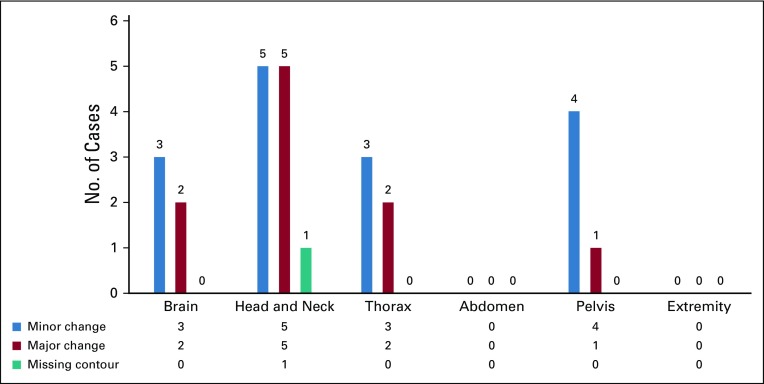
Categorization of changes with respect to treatment site.

**FIG 4:**
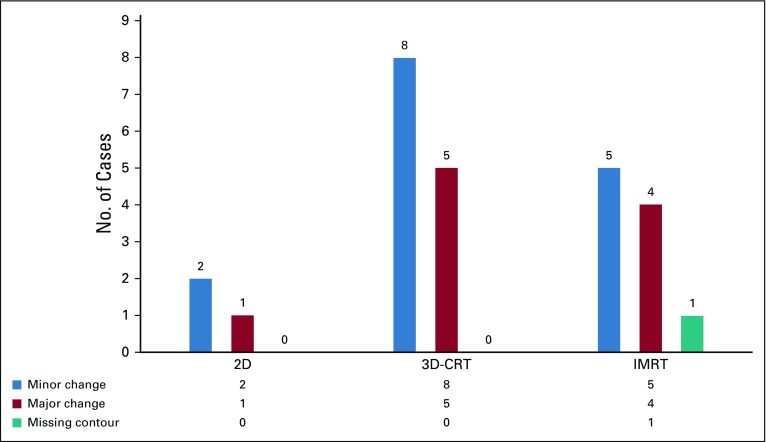
Categorization of changes with respect to treatment technique. 2D, two dimensional; 3D-CRT, three-dimensional conformal radiation therapy; IMRT, intensity modulated radiation therapy.

Radical treatment plans were recommended for the majority of changes as compared with palliative plans, as shown in Fig 2. Among all the treated sites, the most frequent changes were suggested in head and neck tumors (42.3%). The breakdown of change recommendations for all the treated regions is presented in Fig 3. When categorized by radiation planning techniques, 3D-CRT constituted the majority of the changes (50%), followed by IMRT (38.5%). Technique-based recommendations for change are displayed in Fig 4. There were no changes recommended in the contour of organs at risk.

## DISCUSSION

Radiation oncology involves complex sequential processes of consultation, simulation, planning, and delivery of treatment. With the involvement of so many personnel and procedures, each stage is prone to mistakes, which may have a negligible to profound effect on the quality of related outcomes and may eventually compromise patient care. Therefore, to manage these discrepancies, there has been a desperate need for the implementation of a tool or intervention to ensure that patients receive treatment with minimum chances of error that is evidence based and appropriate to their medical condition.^9^ It is imperative to note that AKU is accredited by the Joint Commission International as an academic medical center. The compliance to rigorous standards of patient safety and quality of health care is binding.^10^ Furthermore, accreditation plays an integral role in establishing methods to evaluate compliance with international standards.

QA aimed at improving outcomes and eliminating errors plays a vital role in the field of radiation medicine. Its importance in radiation oncology is even greater because of the unique nature of the multiple phases involved. Quality control indicators are established in an attempt to ensure consistent, safe, and optimal delivery of radiation, meeting the highest standards of care.^5^ Examples of such quality indicators include review of treatment plans and port films by physicians and physicists, chart rounds, and timeouts before treatment delivery.^11^ The quality management guidelines focusing on measuring the functional performance of the radiotherapy equipment by measurable parameters have been provided by the American Association of Physicists in Medicine.^12-15^ The American College of Radiology, the American Society for Radiation Oncology, and the European Society for Radiotherapy and Oncology have also developed practical guidelines to ensure quality control during radiation delivery.^16,17^

Peer review is a valuable tool that is central to QA programs in health care. It is practiced widely among radiation oncologists and has several dimensions, including review of treatment decision making, planning contours, and other parameters including, but not limited to, prescribed dose, target volumes, technique, and patient set-up.^8^ Cancer Care Ontario defines peer review as the evaluation of the components of a radiation treatment plan by a second radiation oncologist.^18^ Peer review is generally the only opportunity for radiation oncologists to evaluate each other’s cases and radiation plans. The input of physicists and radiation therapy technologists is highly valued,^19^ and the Association of Physicists in Medicine has published guidelines for a peer review process between two qualified clinical radiation oncology physicists.^20^ Peer review generally leads to small but potentially significant developments in professional practice and health care outcomes. The success of review and feedback seems to depend on baseline performance and on how the feedback is provided.^21^ This becomes more important in developing countries, which may lack expertise.^22^

There are a number of different formats for conducting peer review. Most centers use peer review in multidisciplinary groups that may or may not have a site-specific focus. The timing of peer review also varies among different centers, with a majority performing peer review before the initiation of treatment.^23^ The concept of site-specific multidisciplinary team meetings, in which physicians from different specialties and other members of the health care team discuss patients with upcoming decision points, is emerging in our country.^24^ However, there is a lack of evidence about formal radiation oncology–specific meetings in Pakistan other than at AKU, where all radiation plans are evaluated by a team of qualified and trainee radiation oncologists.

The goal of highly conformal radiation techniques such as 3D-CRT and IMRT is to increase the likelihood of tumor control by precisely shaping the dose distribution to the target volume. The reliable delivery of high-quality therapy poses a unique challenge. It is essential to account for all possible sources of uncertainties in treatment planning if we are to achieve this goal. It is the preliminary responsibility of the radiation oncologist during treatment planning to precisely delineate the target volumes and normal organs at risk. Inaccurate demarcation may lead to a geographic miss of a high-risk region and hence, reduced local control and poor survival.^25^ This also exposes the surrounding normal tissues to excess doses of radiation beyond their tolerance limits, resulting in higher toxicities. This severity of toxicity warrants the use of precise techniques of delivering radiation.^26^

It is not uncommon to have a disagreement among radiation oncologists about the delineation of target structures, which may invariably have an impact on disease-related outcomes. Interobserver variability in target volumes for cancer treatment with radiation therapy has been demonstrated in multiple studies.^27-29^ Lee et al^30^ performed a study in which they investigated interobserver variation among five radiation oncologists. Each radiation oncologist was asked to contour the prostate independently on axial computed tomography images 1 day after implanting interstitial needles. There were significant interobserver differences in the defined prostate volume, which led to significant differences in dosimetry. Similarly, Logue et al^31^ reported significant interphysician variability in producing target volumes and radiation plans for conformal radiotherapy. These skills are enhanced by knowledge about regional anatomy and patterns of failure; above all, the physician’s experience plays an important role in drawing a CTV.^32^ There is a need to quantify disagreements, and additional effort to increase interobserver agreement is required.

Several techniques and procedures have been developed to overcome this variation. Contrast agents and metallic markers have been used commonly to demonstrate the anatomy during simulation to precisely delineate the target volume and to increase the probability of tumor control.^33^ Valicenti et al^34^ presented a study in which seven radiation oncologists delineated the prostate in groups of patients with and without contrast medium. The study concluded that the use of urethral and bladder contrast improved the reliability of localizing the prostate. We routinely use contrast media and markers as per international recommendations.

Inconsistency in contouring seems to be greater when planning for adjuvant radiation therapy in postoperative cases. The introduction of standardized contouring protocols has led to uniformity in delineation patterns among physicians. In a study by Mitchell et al,^35^ the radiation plans of three patients who had undergone a prostatectomy were contoured by six radiation oncologists before and after, providing a contouring atlas. A significant reduction in variability in target volume outlining was achieved by adhering to an evidence-based contouring protocol. The importance of such protocols has also been highlighted by Goodman et al,^36^ who presented stepwise contouring guidelines and an atlas for the delineation of CTV in the postoperative irradiation of pancreatic cancers. These guidelines have helped physicians better determine areas at risk and minimize dose to normal structures. The planning target volume accounts for the patient set-up error and internal target motion but not for the interobserver variation in defining the CTV.^37^

In our study, 26 plans out of 116 (22.6%) were recommended for changes; this frequency of change is much higher than that reported in contemporary literature.^38-40^ All the recommendations, major or minor, were implemented by the primary radiation oncologist before the radiation treatment plan was delivered to the treatment unit. In 2015, the American Society for Radiation Oncology conducted a nationwide survey related to peer review. The results showed that 83% of radiation oncologists were involved in the process, and among those, 90% changed their radiation plans because of peer review, with approximately 7% to 10% of plans being changed.^38^ Boxer et al^39^ did a prospective real-time audit of 208 patients from June 2007 to June 2008. Eight patients out of the 208 (3.8%) had a change in management strategy recommended. Brundage et al^40^ documented the pretreatment peer review of 3,052 treatment plans over a period of 8 years. Out of the 3,052 audited radiation plans, 124 (4.1%) were identified as having errors in radiation planning or prescription. Modifications were made in 98 plans (3.2%) before commencing radiation. The remainder went unchanged because there were slight deviations from usual practice because of individual patient characteristics.

Delineation of target volume has been identified as the most common variable that is prone to any alteration. In our study, CTV was changed in a total of 19 cases (16.4%), which is consistent with the literature.^39,41^ In the audit performed by Boxer et al,^39^ most of the changes were suggested in target volume coverage (2.9%), as compared with prescribed dose and fractionation schedule. With the use of precise radiation delivery techniques, the chances of marginally missing the target volume are enhanced. Therefore, there is a need to delicately contour the volumes when using highly conformal radiation techniques. A multi-institutional study by Lo et al^41^ reported changes in 66.7% of stereotactic body radiation therapy plans for lung cancer after peer review. The authors of this study concluded that inadequate target volume coverage was the main compulsion behind any change recommendation. Our nascent experience with IMRT has forced us to be extra cautious during contouring and also to seek help from colleagues. Although we most frequently use 3D-CRT in our center, data show that IMRT plans resulted in the most changes in CTV (ie, 10 out of 19).

Our study emphasizes the need for peer review and its impact on the planning process, but several shortcomings require additional attention. First, the sample size was too small to draw any meaningful inferences. Second, we did not compare the pre- and postreview changes in the target volumes. It is the usual practice of our radiation oncologists to make changes in the existing contours. This might be helpful in preventing planning on the wrong target volume, but it does not allow us to quantify the difference in the sizes of altered volumes. Cardenas et al^42^ found that postreview volumes were larger than the prereview ones. Third, in many instances, after a thorough and detailed discussion in SRM, the treatment strategy was completely changed and the patient was referred to a service other than radiation oncology. Therefore, we have routine discussions about the need to give radiation and at which doses, and we make required changes after a consensus is achieved; however, documentation is lacking in this regard. Now, we have started daily peer review meetings, and we plan to publish results of daily peer meetings with an increased sample size and the addition of new variables.

Peer review is one of the most effective ways of dealing with routine but controversial patient-specific decisions in radiation oncology. It provides an opportunity to standardize clinical practice patterns and to develop uniform treatment planning guidelines, with a goal of improved and individualized patient care. Our study has shown that up to a quarter of contours may change with peer review. More work is required in this area for better treatment plans, especially in developing countries.

## References

[1] Contesini M Guberti M Saccani R et al Setup errors in patients with head-neck cancer (HNC), treated using the Intensity Modulated Radiation Therapy (IMRT) technique: How it influences the customised immobilisation systems, patient’s pain and anxiety Radiat Oncol 12 72 2017 28449698 10.1186/s13014-017-0807-y PMC5408424

[2] Segedin B Petric P Uncertainties in target volume delineation in radiotherapy – are they relevant and what can we do about them? Radiol Oncol 50 254 262 2016 27679540 10.1515/raon-2016-0023 PMC5024655

[3] Berry SL Boczkowski A Ma R et al Interobserver variability in radiation therapy plan output: Results of a single-institution study Pract Radiat Oncol 6 442 449 2016 27374191 10.1016/j.prro.2016.04.005 PMC5099085

[4] Radiotherapy CPfQ Quality assurance guidelines for Canadian radiation treatment programs 2015 http://www.cpqr.ca/wp-content/uploads/2013/09/QRT2015-12-03.pdf

[5] Levitt SH Khan F Quality assurance in radiation oncology Cancer 74 2642 2646 1994 7954279 10.1002/1097-0142(19941101)74:9+<2642::aid-cncr2820741810>3.0.co;2-e

[6] The Linux Information Project Peer review definition 2005 http://www.linfo.org/peer_review.html

[7] Jamtvedt G Young JM Kristoffersen DT et al Audit and feedback: Effects on professional practice and health care outcomes Cochrane Database Syst Rev 3 CD000259 2003 12917891 10.1002/14651858.CD000259

[8] Marks LB Adams RD Pawlicki T et al Enhancing the role of case-oriented peer review to improve quality and safety in radiation oncology: Executive summary Pract Radiat Oncol 3 149 156 2013 24175002 10.1016/j.prro.2012.11.010 PMC3808744

[9] Burmeister B Quality assurance in radiation oncology Cancer Forum 36 86 88 2012

[10] Joint Commission International https://www.jointcommissioninternational.org/

[11] Ford EC Terezakis S Souranis A et al Quality control quantification (QCQ): A tool to measure the value of quality control checks in radiation oncology Int J Radiat Oncol Biol Phys 84 e263 e269 2012 22682808 10.1016/j.ijrobp.2012.04.036

[12] Fraass B Doppke K Hunt M et al American Association of Physicists in Medicine Radiation Therapy Committee Task Group 53: Quality assurance for clinical radiotherapy treatment planning Med Phys 25 1773 1829 1998 9800687 10.1118/1.598373

[13] Nath R Amols H Coffey C et al Intravascular brachytherapy physics: Report of the AAPM Radiation Therapy Committee Task Group No. 60 Med Phys 26 119 152 1999 10076966 10.1118/1.598496

[14] Nath R Anderson LL Meli JA et al Code of practice for brachytherapy physics: Report of the AAPM Radiation Therapy Committee Task Group No. 56 Med Phys 24 1557 1598 1997 9350711 10.1118/1.597966

[15] Rivard MJ Coursey BM DeWerd LA et al Update of AAPM Task Group No. 43 Report: A revised AAPM protocol for brachytherapy dose calculations Med Phys 31 633 674 2004[Erratum: Med Phys 31:3532-3533, 2004] 15070264 10.1118/1.1646040

[16] Hartford AC Galvin JM Beyer DC et al American College of Radiology (ACR) and American Society for Radiation Oncology (ASTRO) practice guideline for intensity-modulated radiation therapy (IMRT) Am J Clin Oncol 35 612 617 2012 23165357 10.1097/COC.0b013e31826e0515

[17] Kouloulias VE Poortmans PM Bernier J et al The Quality Assurance programme of the Radiotherapy Group of the European Organization for Research and Treatment of Cancer (EORTC): A critical appraisal of 20 years of continuous efforts Eur J Cancer 39 430 437 2003 12751372 10.1016/s0959-8049(02)00113-2

[18] Cancer Care Ontario Radiation Oncology Peer Review Guidance. 2013 https://www.cancercareontario.ca/en/guidelines-advice/types-of-cancer/3246

[19] Adams RD Marks LB Pawlicki T et al The new radiation therapy clinical practice: The emerging role of clinical peer review for radiation therapists and medical dosimetrists Med Dosim 35 320 323 2010 21055612 10.1016/j.meddos.2010.09.002

[20] Halvorsen PH Das IJ Fraser M et al AAPM Task Group 103 report on peer review in clinical radiation oncology physics J Appl Clin Med Phys 6 50 64 2005 10.1120/jacmp.v6i4.2142 PMC5723459 16421500

[21] Ivers N Jamtvedt G Flottorp S et al Audit and feedback: Effects on professional practice and healthcare outcomes Cochrane Database Syst Rev 6 CD000259 2012 22696318 10.1002/14651858.CD000259.pub3 PMC11338587

[22] Khalid F Abbasi AN Challenges faced by Pakistani healthcare system: Clinician’s perspective J Coll Physicians Surg Pak 28 899 901 2018 30501822 10.29271/jcpsp.2018.12.899

[23] Brundage M Foxcroft S McGowan T et al A survey of radiation treatment planning peer-review activities in a provincial radiation oncology programme: Current practice and future directions BMJ Open 3 e003241 2013 10.1136/bmjopen-2013-003241 PMC3731715 23903814

[24] Abbasi AN Effective cancer management can only be possible via a multi-disciplinary team approach-need for establishment of site specific tumor boards Nat J Health Sci 1 2 4 2016

[25] Jones MP Martin J Foo K et al The impact of contour variation on tumour control probability in anal cancer Radiat Oncol 13 97 2018 29776418 10.1186/s13014-018-1033-y PMC5960192

[26] Jameson MG Kumar S Vinod SK et al Correlation of contouring variation with modeled outcome for conformal non-small cell lung cancer radiotherapy Radiother Oncol 112 332 336 2014 24853367 10.1016/j.radonc.2014.03.019

[27] Senan S van Sörnsen de Koste J Samson M et al Evaluation of a target contouring protocol for 3D conformal radiotherapy in non-small cell lung cancer Radiother Oncol 53 247 255 1999 10660205 10.1016/s0167-8140(99)00143-7

[28] Grills IS Yan D Black QC et al Clinical implications of defining the gross tumor volume with combination of CT and 18FDG-positron emission tomography in non-small-cell lung cancer Int J Radiat Oncol Biol Phys 67 709 719 2007 17197120 10.1016/j.ijrobp.2006.09.046

[29] Teh BS Bastasch MD Wheeler TM et al IMRT for prostate cancer: Defining target volume based on correlated pathologic volume of disease Int J Radiat Oncol Biol Phys 56 184 191 2003 12694837 10.1016/s0360-3016(03)00085-3

[30] Lee WR Roach M III Michalski J et al Interobserver variability leads to significant differences in quantifiers of prostate implant adequacy Int J Radiat Oncol Biol Phys 54 457 461 2002 12243822 10.1016/s0360-3016(02)02950-4

[31] Logue JP Sharrock CL Cowan RA et al Clinical variability of target volume description in conformal radiotherapy planning Int J Radiat Oncol Biol Phys 41 929 931 1998 9652859 10.1016/s0360-3016(98)00148-5

[32] Vinod SK Min M Jameson MG et al A review of interventions to reduce inter-observer variability in volume delineation in radiation oncology J Med Imaging Radiat Oncol 60 393 406 2016 27170216 10.1111/1754-9485.12462

[33] Campostrini F Garusi G Donati E A practical technique for conformal simulation in radiation therapy of pelvic tumors Int J Radiat Oncol Biol Phys 32 355 365 1995 7751177 10.1016/0360-3016(94)00448-T

[34] Valicenti RK Sweet JW Hauck WW et al Variation of clinical target volume definition in three-dimensional conformal radiation therapy for prostate cancer Int J Radiat Oncol Biol Phys 44 931 935 1999 10386652 10.1016/s0360-3016(99)00090-5

[35] Mitchell DM Perry L Smith S et al Assessing the effect of a contouring protocol on postprostatectomy radiotherapy clinical target volumes and interphysician variation Int J Radiat Oncol Biol Phys 75 990 993 2009 19345515 10.1016/j.ijrobp.2008.12.042

[36] Goodman KA Regine WF Dawson LA et al Radiation Therapy Oncology Group consensus panel guidelines for the delineation of the clinical target volume in the postoperative treatment of pancreatic head cancer Int J Radiat Oncol Biol Phys 83 901 908 2012 22483737 10.1016/j.ijrobp.2012.01.022 PMC6211792

[37] Prescribing, recording, and reporting photon-beam intensity-modulated radiation therapy (IMRT). Journal of the ICRU 10:1-106, 2010 10.1007/s00066-011-0015-x 22234506

[38] Hoopes DJ Johnstone PA Chapin PS et al Practice patterns for peer review in radiation oncology Pract Radiat Oncol 5 32 38 2015 25413419 10.1016/j.prro.2014.04.004

[39] Boxer M Forstner D Kneebone A et al Impact of a real-time peer review audit on patient management in a radiation oncology department J Med Imaging Radiat Oncol 53 405 411 2009 19695048 10.1111/j.1754-9485.2009.02096.x

[40] Brundage MD Dixon PF Mackillop WJ et al A real-time audit of radiation therapy in a regional cancer center Int J Radiat Oncol Biol Phys 43 115 124 1999 9989522 10.1016/s0360-3016(98)00368-x

[41] Lo AC Liu M Chan E et al The impact of peer review of volume delineation in stereotactic body radiation therapy planning for primary lung cancer: A multicenter quality assurance study J Thorac Oncol 9 527 533 2014 24736076 10.1097/JTO.0000000000000119

[42] Cardenas CE Mohamed ASR Tao R et al Prospective qualitative and quantitative analysis of real-time peer review quality assurance rounds incorporating direct physical examination for head and neck cancer radiation therapy Int J Radiat Oncol Biol Phys 98 532 540 2017 28258898 10.1016/j.ijrobp.2016.11.019 PMC5438284

